# Improved production of β-carotene in light-powered *Escherichia coli* by co-expression of *Gloeobacter* rhodopsin expression

**DOI:** 10.1186/s12934-023-02212-0

**Published:** 2023-10-10

**Authors:** Chao-Yu Lee, Kai-Wen Chen, Chih-Lu Chiang, Hsuan-Yu Kao, Hao-Cheng Yu, Hsiao-Ching Lee, Wen-Liang Chen

**Affiliations:** 1 Institute of Molecular Medicine and Bioengineering, Department of Biological Science & Technology, National Yang Ming Chiao Tung University, Hsinchu, 300093 Taiwan; 2 Department of Mechanical Engineering, National Yang Ming Chiao Tung University, Hsinchu, 300093 Taiwan; 3 Department of Civil Engineering, National Yang Ming Chiao Tung University, Hsinchu, 300093 Taiwan

**Keywords:** *Gloeobacter* rhodopsin, Rhodopsin, *Escherichia coli*, Phototroph, Biosynthesis

## Abstract

**Background:**

Providing sufficient and usable energy for the cell factory has long been a heated issue in biosynthesis as solar energy has never been rooted out from the strategy for improvement, and turning *Escherichia coli (E. coli)* into a phototrophic host has multiple captivating qualities for biosynthesis. In this study, β-carotene was a stable compound for production in *E. coli* with the expression of four enzymes (*CrtE, CrtB, CrtI, CrtY*) for production due to its light-harvesting feature as an antenna pigment and as an antioxidant and important precursor for human health. The expression of *Gloeobacter* rhodopsin (GR) in microbial organisms was proved to have potential for application.

**Results:**

The expression of fusion protein, GR-GFP, in *E. coli* showed visible GFP signal under fluorescent microscopy, and its in vivo proton pumping activity signal can be detected in induced photocurrent by electrodes on the chip under intervals of illumination. To assess the phototrophic synthesis ability of the host strain compared to wild-type and vector control strain in chemostat batch with illumination, the expression of red fluorescent protein (RFP) as a target protein showed its yield improvement in protein assay and also reflected its early dominance in RFP fluorescence signal during the incubation, whereas the synthesis of β-carotene also showed yield increase by 1.36-fold and 2.32-fold compared with its wildtype and vector control strain. To investigate the effect of GR-GFP on *E. coli*, the growth of the host showed early rise into the exponential phase compared to the vector control strain and glucose turnover rate was elevated in increased glucose intake rate and upregulation of ATP-related genes in glycolysis (*PtsG, Pgk, Pyk*).

**Conclusion:**

We reported the first-time potential application of GR in the form of fusion protein GR-GFP. Expression of GR-GFP in *E. coli* improved the production of β-carotene and RFP. Our work provides a strain of *E. coli* harboring phototrophic metabolism, thus paving path to a more sustainable and scalable production of biosynthesis.

## Background

**Fig. 1:**
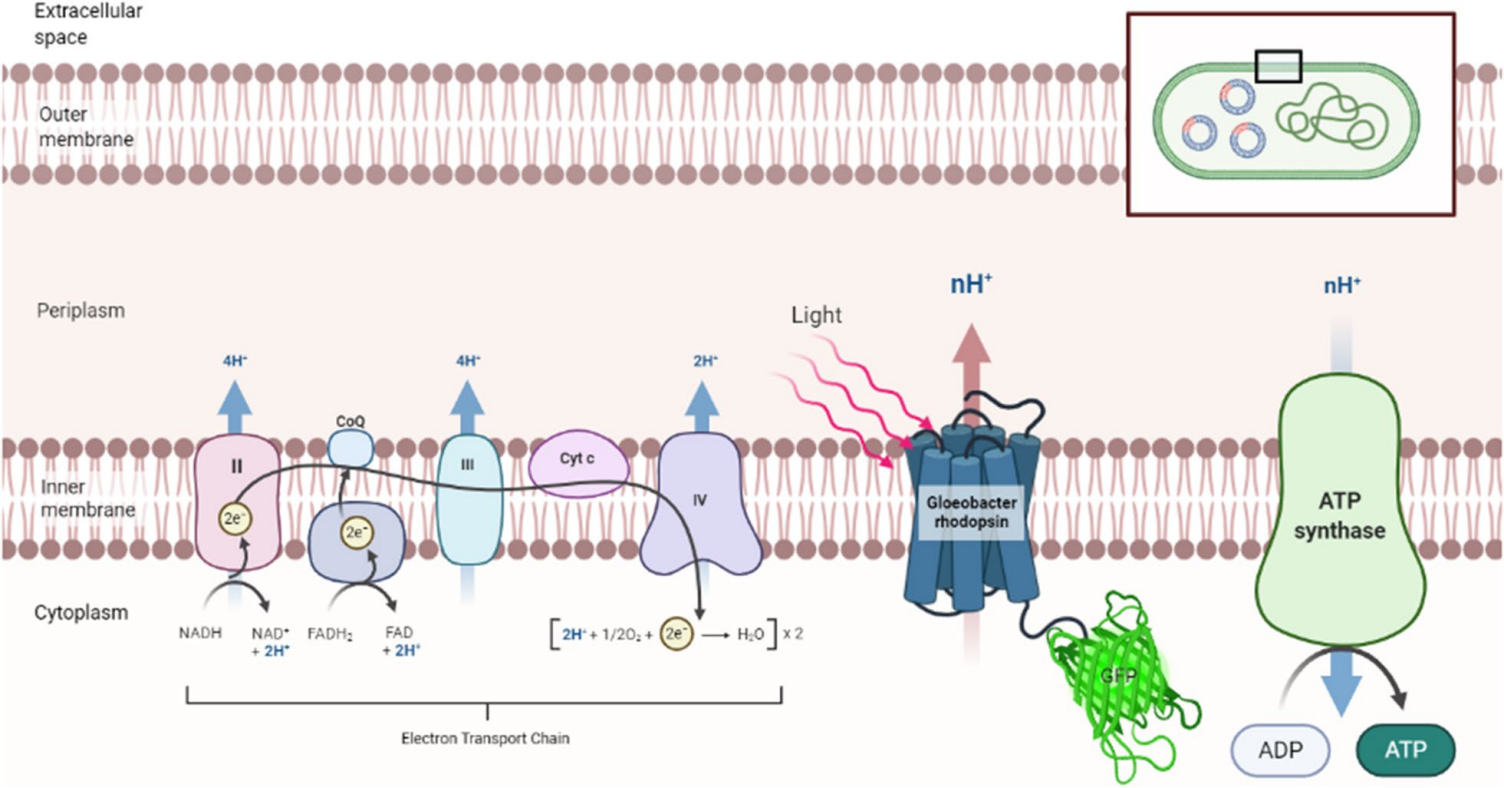
Diagram of the proposed model for GR-GFP in *E. coli* BL21 (DE3)*.* In the chemiosmotic coupling of *E. coli*, we hypothesized that the expression of *Gloeobacter* rhodopsin on the inner membrane could act as an additional source of proton gradient driven by light, thus promoting the proton motive force of ATP synthase. Also, the green fluorescence protein (GFP) was designed as a reporter gene for proper expression and folding of the fusion protein GF-GFP

Renewable energy has gained attention in recent years, as we endeavored to transcend the limitation by incorporating light energy into the cell host. Microbial rhodopsin are noticeable candidates for this notion by directly absorbing light as energy source for proton motive force (PMF) in chemiosmotic coupling, these ancient microbials are able to gain energy that resembled plants (Fig. 1). Accordingly, expressing the functional rhodopsin in *Escherichia coli* could endow the cell with more capacity for secondary active transport [1]. We selected *Gloeobacter* rhodopsin for its independent function of proton pumping activity on the cell membrane and its high turn-over rate compared to proteorhodopsin. *Gloeobacter* rhodopsin (GR) is one of the type I rhodopsins derived from the cyanobacteria, *Gloeobacter violaceus* PCC7421. According to its genome analysis, it diverged early from the lineage of cyanobacteria and developed its independent auto-heterotrophic lifestyle, and *Gloeobacter* rhodopsin is located on its cytoplasmic membrane together with respiratory electron transport chain system instead of chlorophyll membrane or thylakoid membrane. It is notable that GR solely serves as a light-harvesting proton pump that offers a compensation for energy system apart from respiration, both responsible for adenosine triphosphate (ATP) production in *Gloeobacter violaceus*.

At present, researchers have already unveiled the potential of *Gloeobacter* rhodopsin [2]; Pil kim et al. expressed GR in *E. coli* BL21 (DE3), showing that overexpression of GR could harbor excessive ATP, responsible for reactive oxidative species (ROS) and growth retardation [3]. They also indicated that evolutionary optimization in *E. coli* BL21 (DE3) could tackle the predicament of ROS [4]. However, the influence of GR in *E. coli* BL21 (DE3) remained undetermined regardless of its overexpression and its potential in production elusive. Its adapted strain offered positive possibilities for energy production and for improving synthetic yields.

In *E. coli* biosynthesis, two general problems lies in its inability of properly investing nutrition and energy for its survival and target production [5]. First, the target protein encoded by expression vector in *Escherichia coli* poses metabolic burden under strong induction upon regulated promoters, as over-activated recombinant genes may produce aggregates of protein products, thus leading to the toxicity to the host and simultaneously the growth challenges derived from vector itself including retaining resistance gene and DNA production, which are factors to be considered in the cultivation [6]. Second, the downsides of overfeeding nutrition loom as several critical growth challenges have been elucidated in some studies [7], including its limited glucose turnover rate and its impact on transcription regulators, which deteriorates the integrity of its gene-harboring subpopulation in the fed-batch [8]. The use of rhodopsin as a strategy to address the problems focuses on providing additional energy to improve its glucose turnover rate in host and thus more bacterial precursors in compound synthesis. Previous studies of expressing rhodopsin have proved potential for improving the growth of the cell and the enhancement of product [9, 10]. We hypothesized that additional energy provided by rhodopsin expression in the cell host could prevent protein aggregate [11] and promote cell growth. In this research, we also observed the growth dependency of GR-GFP expressed in *E. coli* and also its ATP-related genes in bacterial glycolysis, and the main cause of this research was to evaluate the potential of the fusion protein we designed, GR-GFP, expressed in *E. coli,* and to discover the underlying mechanism of the fusion protein. The co-expression of recombinant genes with GR-GFP were selected for its availability of being easily-observable in the bacterial cell factory. So, we opted for the target protein, RFP, in *E. coli* co-expressing GR-GFP, and expressed four enzymes for β-carotene production. The choice of β-carotene as a production target was important due to its potential property for antenna pigment for the rhodopsin [12] and stable production history [13]. If we were able to find positive results from expressing recombinant protein, we could infer that enzyme expression and its compound synthesis could thus be improved, as we perform stepwise characterizations of GR-GFP.

## Material and methods

### Gene, plasmid and strain construction

GR-GFP fusion protein gene was designed under T7 promoter and synthesized from Genwiz in the plasmid pUC57a, and the gene were further subcloned into expression vector pET32a (Navogen, USA) in the restriction sites, EcoRI and BamHI with a C-terminal 6x-His tag. The cloning process was performed in *E. coli* DH5 alpha, and the resultant plasmid was stored at − 20 °C. The gene and its vector control, pET32a heat-shock transformed *E. coli* BL21 (DE3). The gene harboring *E. coli* BL21 (DE3) were made competent with 100 mM calcium chloride and 20% glycerol mix and stored at − 80 °C. The strains and plasmid used in this study were also listed in Table 1.

**Table 1:** Strains and plasmids used in this study

Strains/plasmids	Genotype	Sources
DH5 alpha	dlacZ Delta M15 Delta(lacZYA-argF) U169 recA1 endA1 hsdR17(rK-mK+) supE44 thi-1 gyrA96 relA1	Lab Stock
Lemo21(DE3)	fhuA2 [lon] ompT gal (λ DE3) [dcm] ∆hsdS/pLemo(CamR) λ DE3 = λ sBamHIo ∆EcoRI-B int::(lacI::PlacUV5::T7 gene1) i21 ∆nin5 pLemo = pACYC184-PrhaBAD-lysY	New England Biolabs
BL21(DE3)	F – ompT hsdSB (rB- mB-) gal dcm (DE3)	Lab Stock
pUC57a	Amp^r^, cloning vector	Genwiz
pET32a	Amp^r^, expression vector	Lab Stock
pSB2K3	Kan^r^, IPTG-inducible vector	iGEM Foundation
pSB4K5	Kan^r^, expression vector	iGEM Foundation

### Fluorescence microscopy of GR-GFP

The expression study of GR in *E. coli* BL21 (DE3) was incubated in Luria–Bertani (LB) Broth. Initially, 5 mL LB broth was set up for incubation as starter unit from colonies of *GR* and its empty vector (pET32a) control. The expression batches in 50 mL LB broth were refreshed with dilution rate of 1/50 with starter unit, and The growing pattern were measured through absorbance at wavelength 600 nm (O.D._600_) with CLARIOstar® Plus (BMG). Once the O.D._600_ reached 0.3, 1 mM of IPTG were added for induction of GR. The batches were incubated at 16 °C for 16 h for membrane expression. The resultant bacteria sample were centrifuged in 1.5 mL microtubes at 4 °C, 6000 rcf, and the process repeated for two times. The pallet were resuspended and washed with 750 μL of phosphate-buffered saline(PBS, 137 mM NaCl, 2.7 mM KCl, 8 mM Na2HPO4, and 2 mM KH2PO4, pH = 7.4). The washed PBS samples were measured with fluorescent excitation wavelength 470 nm, and documented through Blook. 20 μL of the washed sample were put on the slide with cover slip and dry at margin. Slides were put under fluorescence microscopy (Olympics) with bright-field and GFP filter and photographed.

### Photocurrent measurement of GR-GFP

**Fig. 2:**
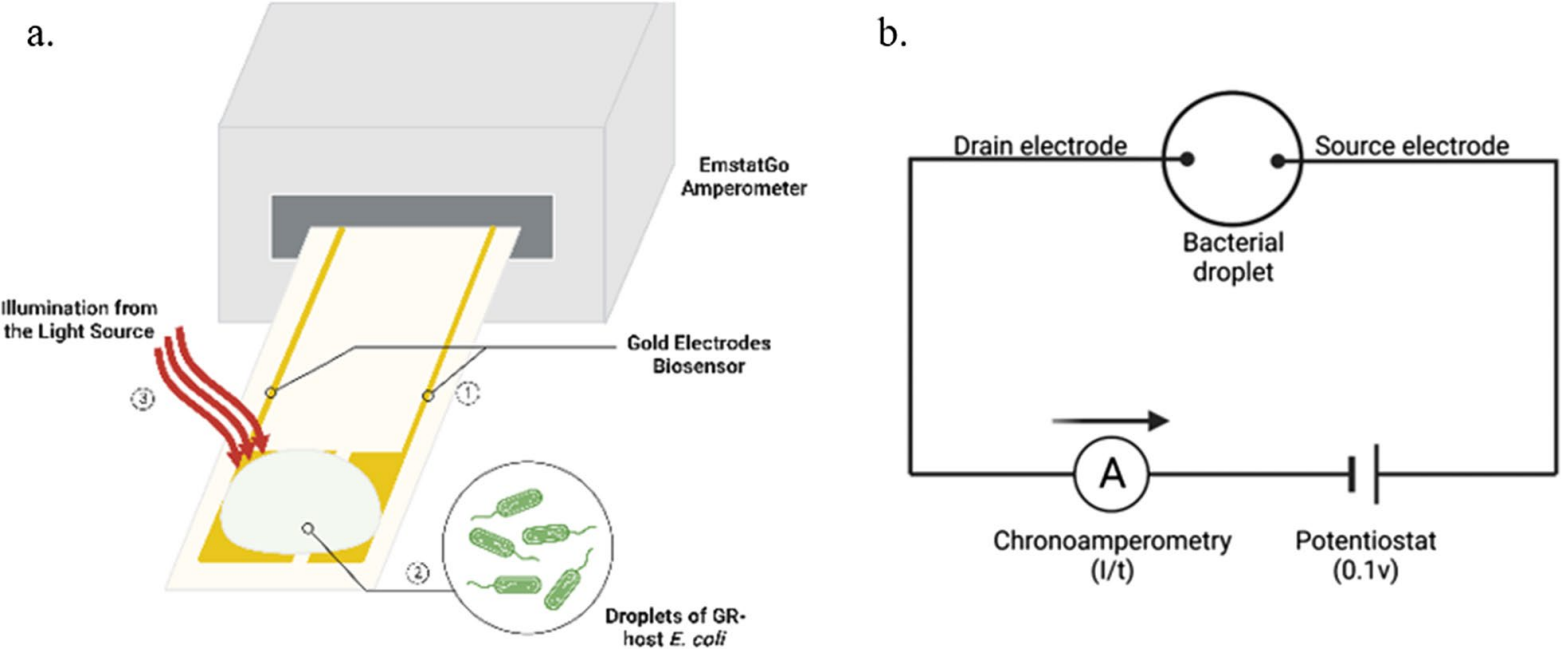
**a** Photocurrent measurement equipment. (①: The electrodes in the chip were connected with the droplet. ②: The droplet contained *E. coli* BL21 (DE3). ③: Upon illumination, the rhodopsin pumped proton into periplasmic space, acidifying the buffer in the droplet). **b** The schematic of biosensor. Under the constant potential (o.1V), electrodes were able to detect the diffused proton pumped by *Gloeobacter* rhodopsin induced by illumination in the droplet, which consisted of *E. coli* BL21 (DE3) and unbuffered saline

GR-GFP-expressing and its vector control (pET32a) *E. coli* BL21 (DE3) from the glycerol stock were revived and incubated in 3 mL LB broth for 16 h. The expression of GR-GFP were performed in 50 mL batch with 1 mL starter unit refresh in 37 °C and incubated for 1 h. The batch were placed on ice for 5 min and IPTG were added to make the final concentration 400 μM, and all-trans retinal to the final concentration of 0.2 mM. The batch were incubated for 24 h in 16 °C for membrane protein expression. The resultant sample were centrifuged 6000*g* for 5 min and washed with unbuffered saline (10 mM NaCl, 10 Mm MgSO4·7H2O, and 0.1 mM CaCl2) twice. The washed sample were normalized with unbuffered saline to O.D.600 to 1.5. 20 μL of bacterial sample were placed on gold thin-filmed electrode chip connecting to potentiostat (EmStatgo) [14] (Fig. 2). The baseline was set up for stabilizing the base line current in dark under 0.1 V potential for 300 s. Intervals of light and dark were 120 s. The first illumination starts after 120 s. Illumination was given four times using flash lights placed above the chip for 3 cm. The photocurrent exited by illumination were documented and the whole measuring process.

### RFP production in GR-GFP host

The gene of red fluorescent protein was derived from iGEM foundation part kit (Biogrick ID: BBa_J04450; parts.igem.org) in plasmid pSB4K5 and pSB2K3 (Table 1). The product plasmid transformed GR-expressing and its vector control *E. coli* BL21 (DE3) competent cell. The inserts of co-expressed colonies were verified with taq-PCR with 2% agarose gel. The RFP of such colonies were incubated in 3 mL LB broth at 37 °C for 12 h and their fluorescence were simultaneously observed. To avoid copy number bias, the bacteria with red fluorescent were chosen for 50 mL as RFP expression batch, in which 400 μM IPTG induction and 0.2 mM all-trans retinal were set up initially, and its growing pattern were observed. The value of O.D._600_ absorbance and florescence at 558 nm were documented every hour. The RFP intensity was normalized with O.D._600_ value, and each data point performed with triplicates. At the end of the incubation, 25 mL of batch sample were collected to 50 mL centrifuge tube for SDS-PAGE protein assay.

### Curcumin synthesis in* Escherichia coli* BL21 (DE3)

Β-carotene synthesis gene cassette (CrtEBIY) was derived from iGEM foundation part kit (Biobrick ID:Bba_K274220; parts.igem.org) in plasmid pSB2K3 under arabinose-inducible promoter (pBAD). The plasmid transformed GR-expressing and empty vector control *E. coli* BL21 (DE3) competent cell and the gene inserts were verified with Taq PCR in 2% agarose gel. The verified colonies were incubated 5 mL LB broth at 37 °C as starter unit overnight and refreshed into 50 mL growth medium (LB broth or M9 medium) until the batch reach O.D.600 to 0.1 for l-arabinose and IPTG induction to make the final concentration 0.1 mM and 0.01 mM and 0.2 mM all-trans retinal. The batches were incubated for 12 h at 37 °C. 25 mL of the batch sample were further centrifuged and the growth medium was discarded and the bacteria pellet was resuspend in 10 mL of PBS, 1mM PMSF and 0.1% Tween 20 and sonicated in 5/25 (sec) on/off with 50% amplitude. The lysate was centrifuged in 5000 rpm for 15 min at 4 °C and the pallet was resuspended with 5 mL 99% acetone to extract β-carotene from the bacteria and subsequently centrifuged in 5000 rpm for 15 min in 4 °C. 1200 μL of acetone supernatant were taken for absorbance at 456 nm wavelength measurement. The sample were placed in 96 well in triplicate and normalized with O.D.600 value from the endpoint of sample harvest. Each sample were done in 5 replicas.

### Growth dependency of GR-GFP host

In characterizing the dependency of growth in GR-expressing *E. coli* BL21 (DE3), the growth during the incubation at 37 °C with constant illumination were documented in 50 mL M9 batch (0.4% glucose), and glucose concentration were measured by 3,5-Dinitrosalicylic acid (DNS) reagent. GR-expressing and its vector control *E. coli* BL21 (DE3) were revived from the glycerol stock into 3 mL LB broth as starter unit in 37 °C overnight. 1 mL of starter units were refreshed into 50 mL growth medium for 1 h and IPTG were added to make the final concentration 400 μM as GR expression batch. The expression batch were incubated in 37 °C for 12 h, whereas the resultant growth medium was discarded after 12,000 rpm centrifugation and washed with equal volume of PBS for one time and centrifuge 12,000 rpm for 30 s under 4 °C. The bacterial sample were normalized to O.D. 600 to 1 with PBS and added into 50 mL growth medium for the second-time incubation. The O.D.600 value were documented every 1 h during the two-time incubation processes.

The treatment of 0.01% sodium azide was performed in the same procedure and was added in the second-time incubation. Every experiment group were measured in triplicates. Standard deviation and growth curve were analyzed with Gompertz model and plotted with GraphPad Prism9.

### Transcriptome profile of GR-GFP host *E. coli* BL21 (DE3)

Total RNA of GR-expressing *E. coli* BL21 (DE3) together with its vector control were collected during the incubation process and its O.D.600 were also documented for reference. Moderate volume of bacterial sample was collected in 50 mL centrifuge tube according to its population and centrifuged in 5000 rpm in 4 °C. The supernatant were discarded and RNAzol® RT (RN 190) were added to extract bacterial RNA according to manufacturer’s instructions. The quality of extracted RNA were evaluated with nanophotometer (Implen) to ensure that O.D.260/O.D.280 > 1.5 and O.D.230/O.D. 280 > 1. 5000 ng of bacterial RNA were reverse-transcripted into cDNA with GScript First-Strand Synthesis Kit (Genedirex) in total volume 20 μL according to manufacterer’s instruction and its quality and concentration were also evaluated with nanodrop. Equal amount of cDNA were diluted with 1/10 ratio and added with its primers and Fast SYBR™ Green Master Mix. Real-time PCR was performed with StepOneTM Real-Time PCR system (Applied Biosystems) with 20 s of 95 °C initial denaturing followed by 40 cycles of 3 s at 95 °C, 30 s at 60 °C and melt curve were also performed at the end of the run. The fluorescent detection was performed in annealing stage and melt curve analysis ensured that single target was amplified. Comparative Ct (delta-delta Ct) was calculated with StepOneTM software v2.3. All qPCR samples were done with triplicates and no template control simultaneously. Relative quantification value were calculated compared with non-treated control and empty vector control. The fold change of genes was plotted with GraghPad Prism9.

## Results

### Fusion protein gene design and visualization of the expression of GR-GFP in *E. coli* BL21 (DE3)

**Fig. 3:**
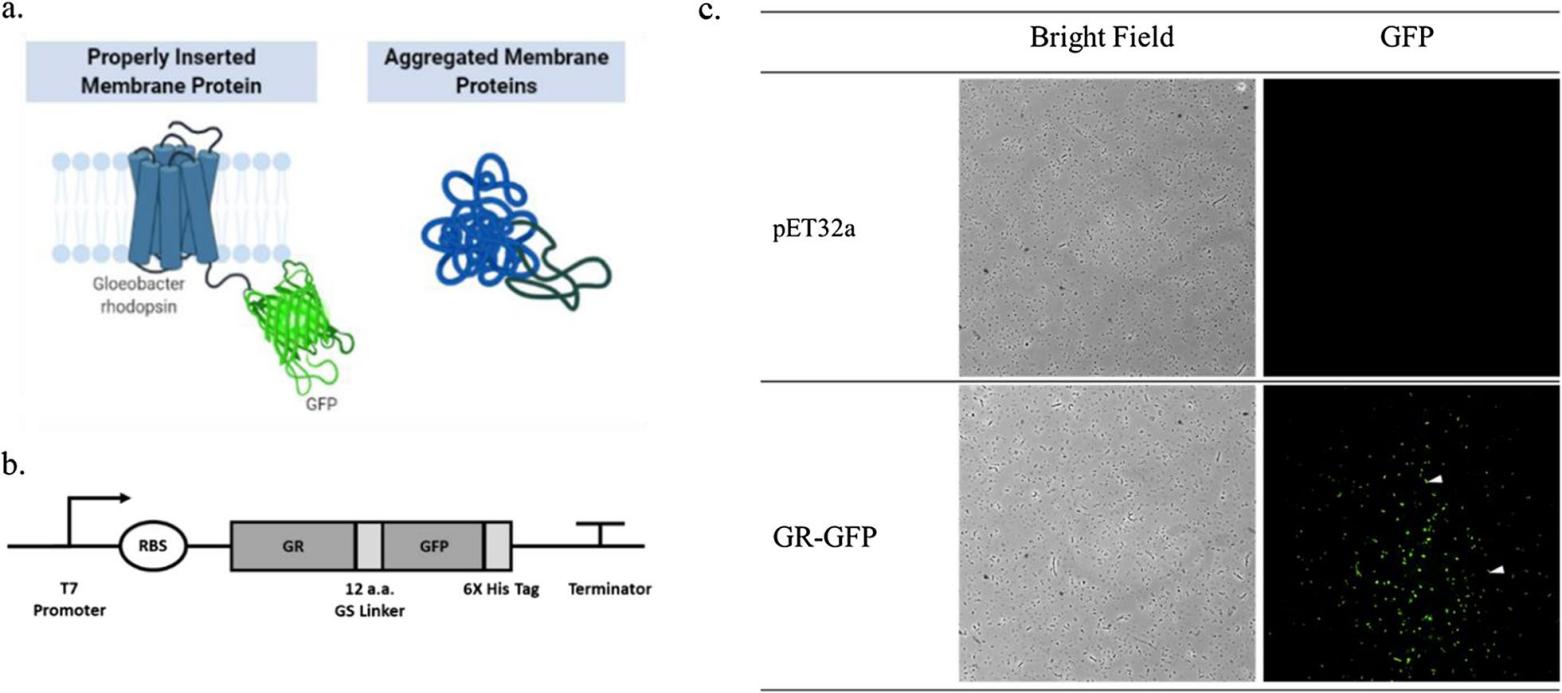
**a** GR-GFP fusion protein model, **b** GR-GFP gene construct, **c** microscopy images (×200) of GR-GFP and its control host. We proposed that GFP acted as a reporter gene to ensure that *Gloeobacter* rhodopsin was expressed with proper folding [15]. The fusion protein (*GR-GFP*) gene were expressed under T7 promoter in expression vector pET32a. Compared with the empty vector control, the green fluorescence could be observed in the host of *E. coli* BL21 expressing GR-GFP (as the white pinnacles demarcated)

The expression of membrane protein with proper folding can be visualized by GFP, as its purpose of fusion protein design (Fig. 3a). Gloeobacter rhodopsin was linked with GFP with a twelve-amino-acid GS linker and regulated by T7 promoter (Fig. 3b). As GR-GFP was expressed in *E. coli* BL21 (DE3), GFP was observable under fluorescence microscopy compared with its vector control, which showed no GFP signal (Fig. 3c). It was noted that the green fluorescence signal was milder than those solely expressed with green fluorescence protein; therefore, under the scope, we observed nearly all *E. coli* glowing with green fluorescence. Also, we found that the expression of GR-GFP can also be observed under blue light exposure after we centrifuged the bacteria in growth medium and washed it with PBS. It could be a preliminary check of the rhodopsin expression for every other experiment in this research.

### Characterization of GR-GFP, the proton pumping activity can be detected by photocurrent on the electrodes

**Fig. 4:**
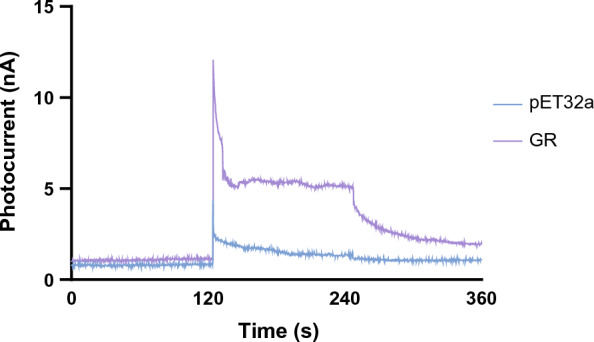
Photocurrent of GR-GFP in *E. coli* BL21 (DE3)*.* Phases of alternating light and dark persisted in every 2 min, as the induction of proton by illumination in GR-host droplet could be observed with the start of illumination at the 120th second to 240 s. Drastic upload of photocurrent was detected at the very start of the illumination and followed with constant plateau of photocurrent, meaning that GR-GFP at first were excited by illumination. The electrodes on the chip directly measured the current in the droplet under constant voltage potential (0.1 v). The measurement was done in biological triplicates and proved to be of the same pattern as the graph showed

Rhodopsin characterization refers to its ion translocation activity. The characterization of GR-GFP in living *E. coli* BL21 (DE3) can be directly measured by its proton pumping activity. The photocurrent will be directly detected by the gold-chip if there is deprotonation happening in the bacteria droplet. To ensure that the proton pumped in the solution was not marred by the buffering effect in the solution, the expressed sample was resuspended and washed in unbuffered saline and placed directly on the electrochemical detection chip to detect the photocurrent induced by illumination. Intervals of light and dark with constant 0.1 V electrical potential were performed. The peak current reflected in GR-GFP-host reflected the deprotonation of its active core in Schiff-base in the traces of illumination and a gradual decay during the dark phase (Fig. 4). The moment the light exposed on the droplet (120th to 240th second), the amperometer showed a sudden and drastic flow of proton, suggesting that GR-GFP was activated and then quenched by the balance of proton through diffusion. In the dark phase after the illumination (240th second to 360th second), it showed a further quench of photocurrent, indicating that the proton pumping activity generated robust photocurrent detectable on biochips. The result showed a sudden and drastic flow of proton initially produced by the start of illumination in GR sample (0.0369 nA/s) compared to the vector control group (0.0067 nA/s), which were consistent with other electrochemical studies of GR [16] and other rhodopsin [17].

### GR-GFP expression improved RFP production

**Fig. 5:**
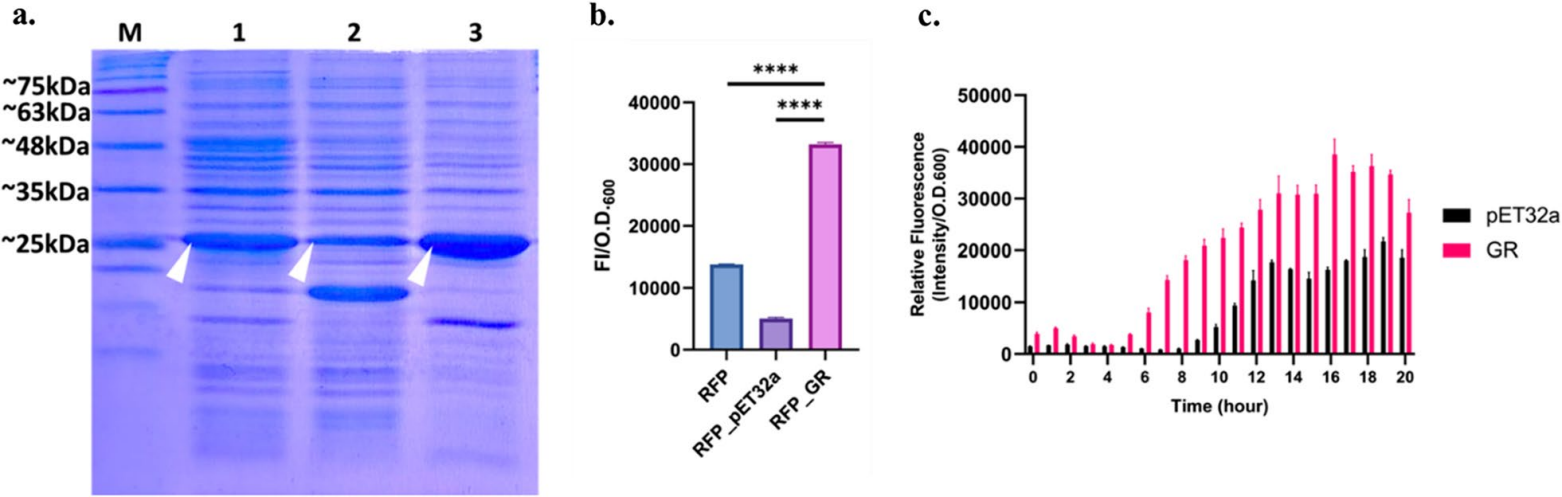
**a** SDS-PAGE of RFP in *E. coli* BL21 (DE3) expressing GR and its controls. (M: blue-elf pre-stained marker, RFP: lane 1, RFP_pET32a: Lane 2, RFP_GR: Lane 3, each well loaded with 16 µg *E. coli* total protein). **b** RFP intensity prior to the protein assay in GR-host and its wildtype control (RFP) and vector control (RFP_pET32a) (****: p-value < 0.0001, done with t-test, triplicate). **c** Growth pattern of RFP intensity in LB batch in GR-GFP host with its vector control (pET32a). Overall, GR-host presented a significant improved of the expression of RFP in its total protein yield and also reflected on its fluorescence intensity during the growth

The focus of this research was to evaluate on the recombinant protein expression in GR-GFP-host by using a common soluble protein RFP as a model protein, as we hypothesized that GR may facilitate the expression of recombinant protein. The choice of RFP as a target protein was conspicuous for its easily-visible quality during the incubation. To eliminate bias in practical manufacturing situation, the control group included wild type *E. coli* BL21 (DE3) and its vector control (pSB4K5). Fluorescent intensity was obtained under the excitation wavelength (558 nm) and normalized with population density (O.D. _600_ value). The production of RFP was analyzed by SDS-PAGE, comparing the total protein distribution in *E. coli* BL21 (DE3)*.* As the hypothesis expected, GR-GFP host showed thicker band over wildtype control and vector control (Fig. 5a). Also, during its incubation, it showed a consistent dependency on GR-GFP that its *E. coli* host showed higher RFP intensity in the 50 mL LB batch (Fig. 5b, c). To observe the difference during the incubation process, the growing pattern of RFP intensity and O.D._600_ in the batch were also documented. The results showed an ample difference among the range from the early rise of RFP signal (Fig. 5c) in GR-expressing host until the stationary phase, suggesting that GR-host can express recombinant protein in its exponential phase.

### GR-GFP expression improved β-carotene production

**Fig. 6:**
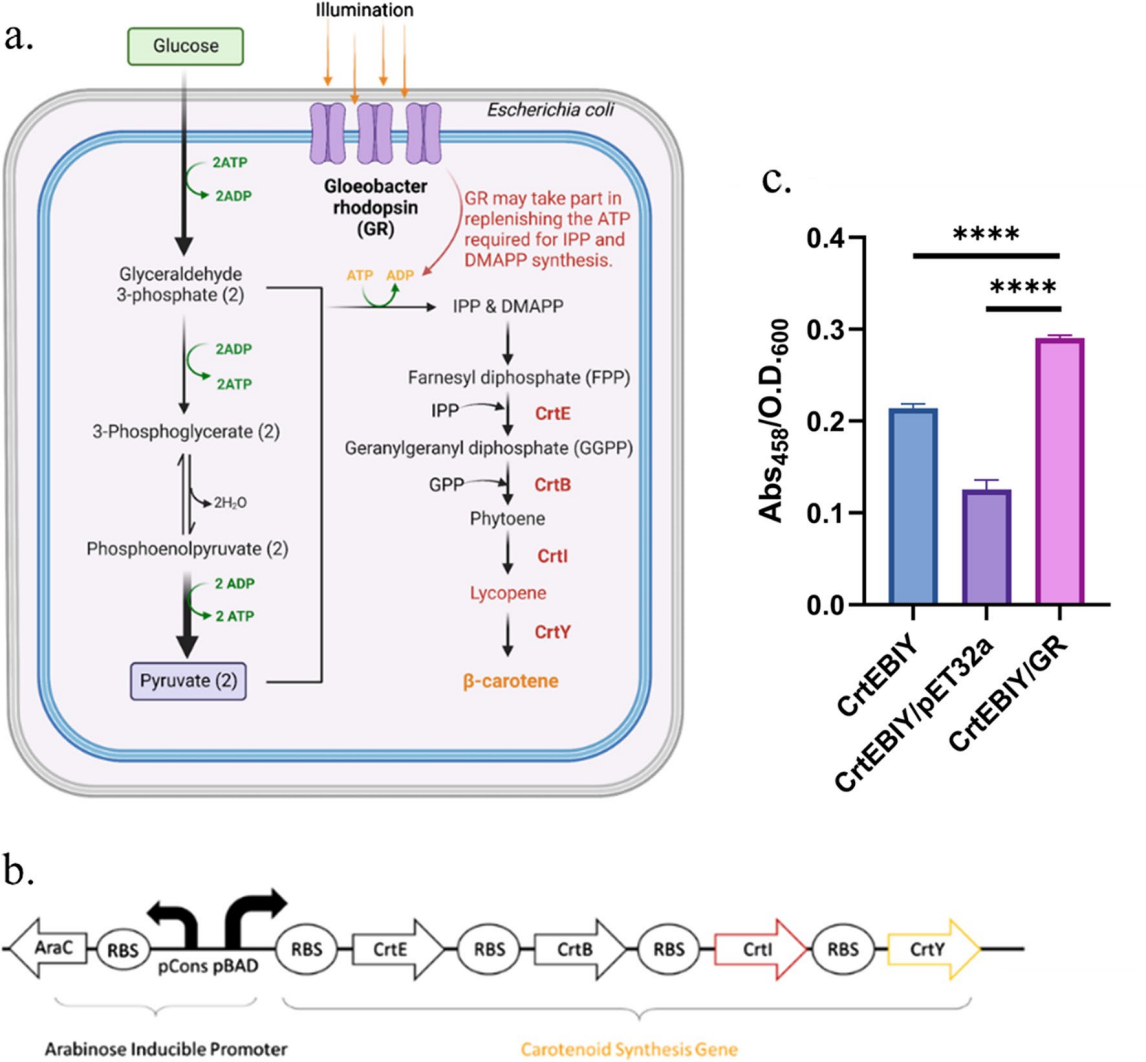
**a** Diagram of the proposed model for β-carotene synthesis in GR-host, **b** gene construct of β-carotene synthesis, which includes *CrtE* (GGPP synthase), *CrtB* (phytoene synthase), *CrtI* (phytoene desaturase), and *CrtY* (lycopene cyclase), all together abbreviated as CrtEBIY. (c) β-carotene production in GR-host and its wildtype control (*CrtEBIY*) and vector control (*CrtEBIY*/pET32a) (****: p-value < 0.0001, done with t-test, average based on five technological repeats). The product of β-carotene was extracted with acetone and measured in the absorbance wavelength (458 nm). The comparison between wildtype control and vector control with GR-host suggested that GR altogether facilitated the synthesis of β-carotene

With the success of expressing the protein targets in GR-host, we intended to confirm that if GR-GFP host would facilitate secondary metabolites synthesis. In this light, we chose a common product, β-carotene as a model molecule for evaluation. To acknowledge the process of β-carotene synthesis, its production pathway in *E. coli* BL21 (DE3) was non-mevalonate pathway, as it preferred to produce isopentenyl diphosphate (IPP) and dimethylallyl diphosphate (DMAPP) through two precursors, glyceraldehyde-3-phosphate (G3P) and pyruvate [13], in which costs ATP for phosphorylation (Fig. 6a). The β-carotene enzymes (*CrtE, CrtB, CrtI, CrtY*) [18] were expressed in GR-host with the gene construct in arabinose promoter in pSB4K5 expression vector (Fig. 6b) (Table 1). The synthesis of β-carotene was incubated in 50 mL LB batch to create an aerobic growing condition in 37 °C with wildtype and vector control. The production of β-carotene was quantified through acetone extraction and absorbance at 456 nm and normalized with its population density. Confirmed from the normalized absorbance intensity, the synthesis in GR-GFP host facilitated the production of β-carotene by 1.36-fold and 2.32-fold in wildtype control and vector control (Fig. 6c).

### *E. coli* BL21 (DE3) expressing GR-GFP shows accelerated growth and consumption of glucose

**Fig. 7:**
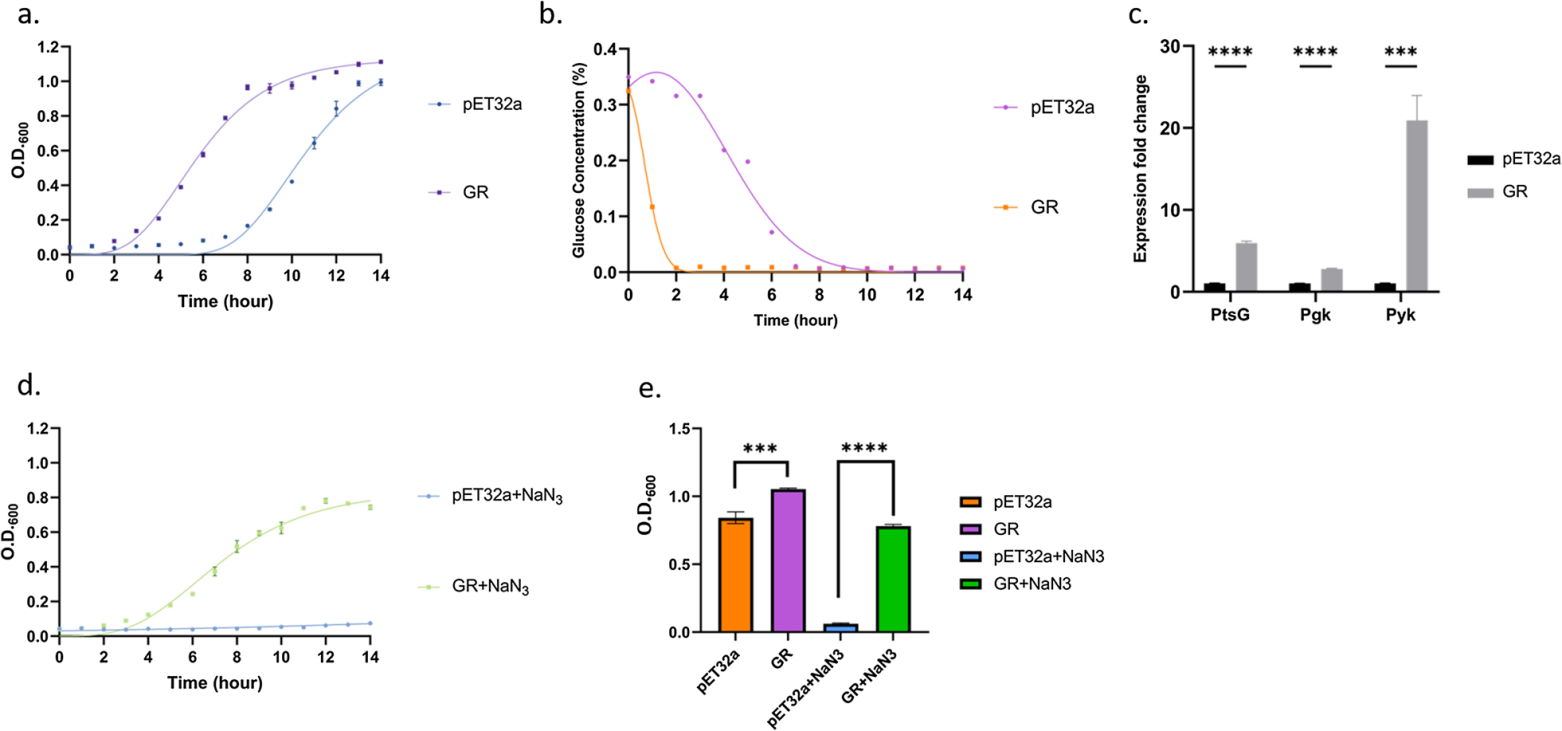
**a** Growth dependency of GR-GFP in M9-glucose medium, **b** glucose consumption of GR-GFP host in M9-glucose medium. **c** transcription fold change of ATP-associated genes in glycolysis in GR-GFP host, **d** growth dependency of GR-GFP under sodium azide salt stress, **e** population comparison of M9 resultant batch. The overall cell physiology in GR-host were evaluated and showed that GR-GFP had an accelerated metabolism that reflected on its growth and with rapid intake of glucose. It was also noteworthy that GR-GFP was able to sustain the growth of *E. coli* in the presence of cytochrome c inhibitor, sodium azide, which proved GR-GFP could be an independent source of PMF for energy generation and cell survival

Whether the PMF exerted by the light-induced proton pump and its theoretical rise in ATP/ADP ratio would reflect on *E. coli* BL21 (DE3) growth and population mass was the next focus in this research. To elucidate the glucose metabolism in GR-GFP host, batch incubation in M9 medium with glucose as carbon source was set up and inoculated with the induced *E. coli* BL21 (DE3) in the presence of IPTG, and its growth pattern and glucose concentration were documented to observe its growing dependency. We found out that GR-GFP expression in *E. coli* BL21 (DE3) had an early entrance into exponential phase compare with the empty vector control, with a slight faster growth rate (0.188 O.D.600*h^−1^ in GR host and 0.180 O.D.600*h^−1^ in pET32a control), and a more rapid consumption of glucose by 2.9-fold, indicating GR-GFP expression offered growing niche over its control with its advantage on glucose consumption (Fig. 7a, b). The finding was consistent with the fact that ATP-enhancement in the host would accelerate the metabolic rate and reflected on its growth. Concordantly, the phenomenon of GR showing better intake of glucose reflects that GR accelerated the cell’s carbon metabolism, which was consistent with the result in *PtsG* expression fold increase (Fig. 7c). It showed that GR-GFP expression activated the bacteria phosphotransferase system (PTS system), and altogether boosted the growth rate of *E. coli* BL21 (DE3). Also, the mass of the population increases slightly compared with the vector control group (Fig. 7e). The phenomenon was observed in more than three biological triplicates using M9 medium and LB broth.

### GR-GFP substitutes electron transport chain as an alternative for chemiosmotic coupling

To prove that the growth dependency could be solely attributed to the PMF exerted by *Gloeobacter* rhodopsin*,* the addition of sodium azide could root out the PMF and terminate oxidative phosphorylation, since sodium azide inhibits cytochrome C oxidase [19, 20]. If *E. coli* BL21 (DE3) harboring GR-GFP survives the salt stress, it would prove that GR exerted its own PMF independently and sustain the growth of its host. The growth of GR-expressing *E. coli* BL21 (DE3) was observed in the presence of 0.001% sodium azide in 50 mL M9 medium with its growth documented every hour. During the incubation process, GR-GFP host treated with sodium azide survived the stress and its growth was also marred by the toxicity of sodium azide compared with the non-treated GR-GFP host, and under the same growing condition, the vector control group all died (Fig. 7d). The O.D. 600 value at 12th hour showed the significance over the treated host and the non-treated control (Fig. 7e). It showed that GR could independently sustain the growth of *E. coli* BL21 (DE3) as electron transport chain was inhibited*.*

### *GR-GFP* activates the transcription of ATP-related genes in glycolysis

Considering the growth pattern and consumption of glucose in *GR*-expressing *E. coli* BL21 (DE3), we hypothesized that *GR* may boost the expression of glycolytic flux; especially in ATP-dependent steps. Therefore, gene expression pattern of *GR* host regarding glycolysis genes (*ptsG, Pgk, Pyk*) were evaluated by real-time PCR. The gene expression fold changes of *GR* host were compared with the vector control group in exponential phase, with *E. coli* BL21 (DE3) house-keeping gene *rrsA* chosen as reference gene [21]. The result (Fig. 7c) showed that the GR-expressing *E. coli* BL21 (DE3) showed robust fold changes in all the three genes (*pstG*: 5.95-fold, *Pgk*: 2.79-fold, *Pyk*: 20.92-fold), especially in pyruvate kinase, indicating that higher growth rate of *GR*-expressing *E. coli* BL21 (DE3) concurred with highly-activated glycolysis (Fig. 7b).

Phosphotransferase system (PTS system) is a unique and major sugar uptake system for *E. coli* BL21 (DE3) using the phosphate energy from phosphoenolpyruvate [22]*,* and *PtsG* (PTS system glucose-specific EIICB component) is a phosphotransferase locating on the inner cell membrane of *E. coli* BL21 (DE3)*,* which transport glucose into the cell and concomitantly phosphorylates glucose to glucose-6-phosphate [23]. It is note-worthy to investigate whether *GR* could exert any influence on PTS system, as cells tend to accumulate sugar inside the cell in the first three steps of glycolysis, which, in turn, consumes ample energy [24]. The result suggested that sugar uptake was hence activated by *GR* expression. Another gene, pgk (phosphoglycerate kinase), is a phosphoglycerate kinase that turns 1,3-biphosphoglycerate into 3-phosphoglycerate (3PG) in the middle of glycolysis. The step garners two ATP molecules from glucose and thus important for carbohydrate utilization in both glycolysis or gluconeogenesis [25]. *GR* expression upregulated *pgk*, which also facilitated the production of ATP. Finally, *pyk* (pyruvate kinase) participates in turning phosphoenolpyruvate into pyruvate, which, in turn, garners ATP. *GR* expression in *E. coli* BL21 (DE3) highly upregulated pyk, which implied the activated formation between phosphoenolpyruvate and pyruvate.

## Discussion

In this research, we hypothesized that expressing the fusion protein (GR-GFP) could benefit the overall physiological condition of growing *E. coli* BL21 (DE3) host and improve its recombinant protein production and compound synthesis. The prove of concept was done stepwise. First, by observing the GFP under microscopy, we could confirm that *E. coli* successfully expressed GR-GFP in the cell. The rhodopsin function of GFP was characterized with illumination on the electrodes of biochip, we detected the photocurrent excited by the illumination. GR-GFP gene in the vector can be successfully expressed on *E. coli* with proper folding and proton pumping activity. Second, we further demonstrated that *E. coli* BL21 (DE3) expressing GR-GFP improved the expression of RFP expression during the process of batch incubation. It was noted that early manifestation of RFP signal pinpoint the fine balance of cell growth and recombinant protein expression with the aid of GR-GFP, although the mechanism contributing to the improvement of protein production remain elusive. It was noticeable that RFP was observable in the early phase of incubation in the batch, indicating that GR-GFP could create a suitable environment for production of recombinant protein in earlier cytoplasm than its control groups. The finding necessitated the need for further discovery into the enzymatic production in the cell, we also proved that GR-GFP improved the production of β-carotene compared with its vector control and wild type control in 50 mL LB batch. Based on the production result, we confirmed that GR-GFP had a positive effect on *E. coli* BL21 (DE3) physiology by providing additional PMF, as it could be potent to sustain the growth of *E. coli* BL21 (DE3) substituting the function of oxidative phosphorylation. This not only proved independent function of GR-GFP in *E. coli* BL21 (DE3) but also offer its potential in turning *E. coli* BL21 (DE3) into a phototrophic strain.

As we proved GR-GFP to be a beneficial gene for improving *E. coli* BL21 (DE3) production yield, our research into unveiling *E. coli* BL21 (DE3) possible dependency on GR-GFP was done in analyzing its relationship in glycolysis and its growth. Previous research showed that GR had retarded growth due to its excessive production of ATP, causing oxidative stress, and it also indicated that by evolutional selection in refreshing batches, *E. coli* BL21 (DE3) could be accommodated to rhodopsin, resulting in accelerated growth. We confirmed that GR-GFP expressed in *E. coli* BL21 (DE3) shared the same tendency with the previous research result [3], as we observed the growth curve on *E. coli* BL21 (DE3) that had fully expressed with GR-GFP with IPTG induction (Fig. 7a). As the main obstacle of chemostat batch in fermentation focus on feeding *E. coli* BL21 (DE3) with glucose to raise its cell density, its glucose turn-over rate was also a critical point for research. We proposed that GR-GFP could re-adjust the cell to an activated state of energy usages as we studied the gene transcription of ATP-associated gene in cell’s exponential phase and proved the activated genes to have ample fold change in GR-GFP. The result was in line with the more activated intake of glucose observed in GR-GFP host (Fig. 7b).

## Conclusion

It is noteworthy that energy refurbishment for biosynthesis and cell survival plays an integral part in metabolic engineering. The motive of expressing rhodopsin in *E. coli* remained promising for strategy of production improvement. In this research, *Gloeobacter* rhodopsin proved itself to have functional proton pumping activity and facilitated the growth and production of the cell factory in the form of fusion protein with GFP. The fusion protein enhanced the expression of recombinant protein RFP and β-carotene with the enzyme cassette (*CrtEBIY*). We offered a promising gene, GR-GFP, for biosynthetic applications. In this research, we co-expressed GR-GFP with expression vector (pET32a) with recombinant protein target in expression vectors harboring kanamycin resistance gene, and still gained the positive niche from GR-GFP. Future researchers could focus on the holistic view of GR-GFP’s mechanism in phototrophic *E. coli* and its application on difficult product for biosynthesis.

## Data Availability

All the data for the proof of the concept in this study were included in this manuscript.

## Abbreviations

GR: *Gloeobacter rhodopsin*
GFP: Green fluorescent protein
RFP: Red fluorescent Protein
GR-GFP: The fusion protein of gloeobacter rhodopsin and green fluorescent protein
IPTG: Isopropyl β-d-thiogalactoside
IPP: Isopentenyl pyrophosphate
DMAPP: Dimethylallyl pyrophosphate
SDS-PAGE: Sodium dodecyl sulfate polyacrylamide gel electrophoresis
